# Inebriety

**Published:** 1890-04-19

**Authors:** 


### The Practitioner's Mirror and Retrospect.

[*The Editor will be glad to receive offers of co-operation and contributions from members of the profession. There is no doubt that much valuable material full of interest and instruction is at present lost to science. This is largely so in the case of* (1) *the younger and less-known members of the hospital staff* (2) *those connected with cottage hospitals, and* (3) *the great body of medical practitioners not attached to any institution. The co-operation of all is cordially invited to enable the Editor to make* The Hospital *of the utmost possible use and value to the profession. Specialists willing to review books on their special subjects are invited to communicate this fact at once. All letters should be addressed to* The Editor, The Lodge, Porchester Square, London, W.]

### MEDICINE.

### INEBRIETY.

Most medical men are frequently called upon to face and treat the effects of inebriety. Mr. Armstrong Willis, of Monmouth, has an article on this subject in the *Pro. Med. Journal*, and he thinks that the strong opinions held on the transmission of a constitutional drink tendency by heredity would justify any amount of plain speaking between doctor and intemperate patient, even when that patient's habits may not have transgressed the limits of social decorum or conventional moderation. For there is more than the recognised facts of inebriety being a predisposing cause for the insanity of children, and of diseases transmitted by alcoholics to their children. It is but necessary to name alcoholic phthisis, hereditary alcoholic gout and rheumatism, and, most characteristic of all, alcoholic cirrhosis and alcoholic contracted kidney. Society is suddenly scandalized out of its usual state of torpid toleration by some strange criminal aberration or monstrous misconduct of one of its members. "Ten to one," writes Mr. Willis, "that there is some history of intemperance behind it." The great aim should be to induce patients to become total abstainers. But to do this it appears to us that the doctor should be a total abstainer also; and we have not surely yet arrived at that extent of self-immolation when we are called upon to become total abstainers for the sake of our patients. Many means are advocated as reliefs and assuagers to the drink crave, such as preparations of bark, acids, sweets, cocoa, fruits, &c.; but the great thing is, if possible, to occupy the mind and exercise the body. An active brotherhood, engaged in manufacturing or agricultural pursuits, would seem to be an ideal establishment where the intemperate could be under restraint, "with a band of earnest Christian workers." Doctors as individuals may, however, do still more. Women recovering from confinements may be warned against the false strength derived from stimulants. Parents may be induced to bring up their children without stimulants. In the serious moments of convalescence, many a chance presents itself much oftener to the doctor than to the cleric to speak a word in favour of abstinence. "The successful treatment of one inebriate, and the restoration of him to a life of sobriety and sanity, may well give a man a greater thrill of joy and pleasure than the achievement of some special triumph of surgery, or the addition of one's name to the roll-call of scientific discoveries." Now it is easy to see that Mr. Willis is an enthusiast in the cause of temperance. Notwithstanding that, from the days of Plutarch, it has been held that heredity is a great cause of drinking-craving we do not know that this has even been unmistakably demonstrated. The author says that Dr. Fletcher Beach, medical superintendent of the Darenth Asylum, showed 31 per cent. of idiotic children to be the off-spring of intemperate parents. But this is not a universal law, and a state of drunken debauch is not "drink-craving." Then, again, we doubt very much if there are really any such hereditary maladies as alcoholic phthisis, alcoholic gout and rheumatism, or alcoholic cirrhosis, and alchoholic contracted kidneys. It appears to us that example during childhood has much more to do with alcoholic craving than heredity. And as regards the cure of the victim who has once developed drink craving, we believe the cure of such a one to be rare indeed. Dr. Willis tells us not to despair, and he says, "Whoever needs hope and encouragement in helping a fellow creature out of the clutch of intemperance, and is inclined in a moment of weariness and apparent failure to give it up, had better read over George Eliot's story of 'Janet's Repentance.'" It is said that "Truth is stranger than fiction," but sometimes fiction is stranger than truth. We do not say that drink-craving is never recovered from; but we say that it is rarely recovered from without personal restraint. And we do not think that the doctor, even although a total abstainer himself, can exert that influence in such cases by "moral suasion," of which Mr. Willis seems to think doctors capable.

